# Diet Quality, Dietary Inflammatory Potential, and All‐Cause Mortality in U.S. Adults With Asthma‐COPD Overlap

**DOI:** 10.1002/fsn3.71657

**Published:** 2026-07-08

**Authors:** Jixiang Li, Jia Yi, Jingxian Tang, Tong Feng, Yuan Wang, Rongrong Guo

**Affiliations:** ^1^ Geriatrics Department The Second People's Hospital of Meishan City, Renshou County People's Hospital Meishan People's Republic of China; ^2^ Department of Respiratory and Critical Care Medicine, the Second People's Hospital of Meishan City Meishan People's Republic of China; ^3^ Department of Respiratory and Critical Care Medicine, Deyang People's Hospital Deyang People's Republic of China; ^4^ Department of Oncology West China Hospital Sichuan University Jintang Hospital, Jintang First People's Hospital Chengdu People's Republic of China

**Keywords:** asthma‐COPD overlap, diet, dietary inflammatory index, healthy eating index, mortality, NHANES

## Abstract

Asthma‐COPD overlap (ACO) is associated with increased mortality and disease burden compared to asthma or COPD alone. Diet, a modifiable risk factor, influences inflammation and lung health, yet its impact on ACO mortality remains understudied. This study investigates the synergistic effects of the Healthy Eating Index (HEI‐2015) and Dietary Inflammatory Index (DII) on all‐cause mortality in ACO patients. Using data from the National Health and Nutrition Examination Survey (NHANES) 2007–2018, we analyzed 609 U.S. adults with ACO, defined by clinical and spirometric criteria. Dietary patterns were assessed via HEI‐2015 (diet quality) and DII (inflammatory potential). Cox proportional hazards models evaluated associations with mortality, adjusting for sociodemographic, lifestyle, and clinical confounders. Restricted cubic spline analyses explored non‐linear relationships, and Least Absolute Shrinkage and Selection Operator (LASSO) regression identified key dietary components for a prognostic nomogram. Healthier diets (higher HEI‐2015) were associated with lower mortality risk (HR = 0.98, 95% CI: 0.97–0.99, highest quartile), while pro‐inflammatory diets (higher DII) increased risk (HR = 1.13, 95% CI: 1.04–1.22). The combination of healthy and anti‐inflammatory diets showed the strongest protective effect (HR = 0.68, 95% CI: 0.47–0.98). LASSO regression identified PUFA, total dairy, whole fruit, and n‐6 fatty acids as key predictors, incorporated into a nomogram with moderate predictive accuracy (AUC: 0.64–0.67). Kaplan–Meier curves confirmed better survival in low‐risk dietary groups (*p* < 0.0001). Subgroup analyses showed stronger effects in mild drinkers and non‐hypertensive patients. High‐quality, anti‐inflammatory diets synergistically reduce mortality in ACO patients. Targeted nutritional interventions emphasizing whole fruits, dairy, and healthy fats may improve outcomes. Future research should validate these findings through interventional trials.

## Graphical abstract

In U.S. adults with asthma‐COPD overlap (ACO) from NHANES 2007–2018, higher Healthy Eating Index‐2015 (HEI‐2015) scores (indicating better diet quality) and lower Dietary Inflammatory Index (DII) scores (indicating anti‐inflammatory diets) were independently and synergistically associated with reduced all‐cause mortality risk. The combination of high HEI‐2015 and low DII conferred the strongest protection (HR = 0.68, 95% CI: 0.47–0.98). Key dietary components (PUFA, total dairy, whole fruit, n‐6 fatty acids) were identified via LASSO regression and incorporated into a prognostic nomogram with moderate predictive accuracy.

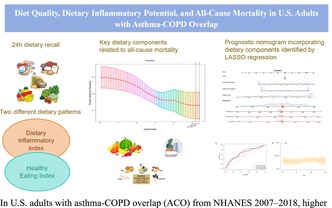

## 1 Introduction

Asthma‐COPD overlap (ACO) is a clinical syndrome that presents with overlapping features of both asthma and chronic obstructive pulmonary disease (COPD). It has drawn growing interest from both researchers and clinicians in recent years. Systematic reviews and meta‐analyses indicate that ACO affects approximately 2.0% of the general population, with significantly higher prevalence among specific clinical groups—reaching up to 26.5% in individuals diagnosed with asthma and 29.6% among those with COPD (Hosseini et al. 2019; Diaz‐Guzman et al. 2011; de Marco et al. 2013). These figures highlight the considerable epidemiological relevance of ACO, particularly among older adults and long‐term smokers, in whom the syndrome is more prevalent and clinically significant due to age‐related lung function decline and cumulative exposure to tobacco smoke (Fouka et al. 2022). The clinical significance of ACO is largely attributable to its substantial disease burden and poorer health outcomes. Patients with ACO tend to experience more frequent exacerbations, accelerated lung function decline, and increased mortality when compared to those with asthma or COPD alone (Leung and Sin 2022; Freiler 2015). Moreover, ACO is associated with worse quality of life, greater impairment in pulmonary function, and higher consumption of healthcare services, culminating in elevated medical expenditures (Kim et al. 2017).

Diet is a well‐established modifiable risk factor in the onset and progression of numerous chronic diseases (Singh et al. 2017; Tosefsky et al. 2024). It not only mitigates the impact of air pollution on lung health (Whyand et al. 2018) but also exerts a protective effect against lung function decline (Zhai et al. 2018). Although direct research on diet and ACO is limited, information from dietary studies on asthma and COPD provides valuable clues for developing dietary interventions in ACO. For instance, research has shown that higher diet quality is associated with reduced levels of inflammatory biomarkers, which are critical in asthma pathophysiology (Nygaard et al. 2021; Koç et al. 2024). Similarly, dietary patterns rich in antioxidants and anti‐inflammatory compounds, such as the Mediterranean diet and the Dietary Approaches to Stop Hypertension (DASH) diet, are consistently linked to improved lung function and a lower risk of COPD development (Scoditti et al. 2019). A comprehensive systematic review and meta‐analysis further corroborated these findings, demonstrating that adherence to healthy dietary patterns—characterized by high consumption of fruits, dietary fiber, and fish—reduces COPD risk by 12% (Parvizian et al. 2020). Recent evidence also suggests a positive correlation between the Dietary Inflammatory Index (DII) score and ACO risk (Wang et al. 2024).

Although previous studies have explored the associations between the Dietary Inflammatory Index (DII) or related indices (e.g., CDAI, PHDI) and ACO risk/prevalence, or the link between dietary inflammation and all‐cause mortality in COPD alone, these studies have several key limitations: most focus on ACO incidence/prevalence or COPD prognosis, with a lack of longitudinal mortality analyses specifically in the ACO overlap phenotype (Wei et al. 2025; Han et al. 2025); few have jointly evaluated overall diet quality (HEI‐2015) and inflammatory potential (DII) to uncover potential synergistic effects (Tian et al. 2024; Gong et al. 2025; Zhao et al. 2025); most remain at the level of overall dietary scores without component‐level selection or development of clinically applicable prognostic tools (Wei et al. 2025; Tian et al. 2024); and some suffer from single 24‐h dietary recall (Wei et al. 2025), less stringent diagnostic criteria (Wei et al. 2025), or inadequate adjustment for weighting/covariates (Zhao et al. 2025).

To address these gaps, the present study, using National Health and Nutrition Examination Survey (NHANES) 2007–2018 data, is the first to: (1) systematically examine the independent and joint effects of HEI‐2015 and DII on all‐cause mortality in confirmed ACO patients (using the average of two 24‐h dietary recalls for greater reliability); (2) construct four dietary pattern groups via median split to clarify whether the healthy + anti‐inflammatory combination exerts the strongest synergistic protective effect; (3) apply LASSO regression to identify dietary components most strongly associated with mortality risk (polyunsaturated fatty acids, total dairy, whole fruit, n‐6 fatty acids); and (4) develop and internally validate a prognostic nomogram (including time‐dependent AUC and Kaplan–Meier curves) to provide a practical clinical tool for individualized mortality risk stratification and targeted nutritional interventions in ACO patients.

## 2 Materials and Methods

### 2.1 Study Design and Population

The NHANES serves as a continuous, nationally representative epidemiological study. Its primary objective is to conduct a thorough evaluation of the health and nutritional conditions of the civilian, non‐institutionalized population in the United States. NHANES employs a stratified, multistage probability sampling method to collect nationally representative data, encompassing demographic characteristics, dietary intake, anthropometric measurements, laboratory tests, health behaviors, and disease conditions. Initiated in 1971, the survey was initially conducted in phases and transitioned to a continuous survey format in 1999, with data released in 2‐year cycles, widely utilized in public health research, policy development, and disease prevention.

**FIGURE 1:**
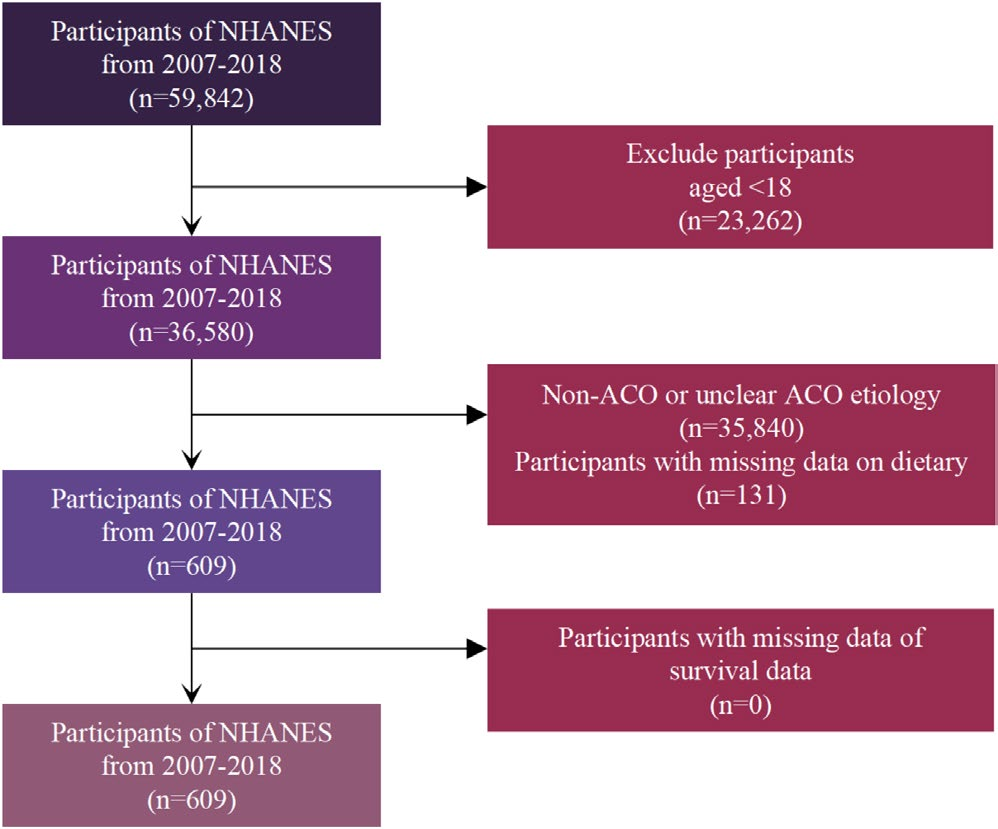
Flowchart for selecting eligible study participants.

The study included adult participants from the NHANES 2007–2018 cycles who completed 24‐h dietary recall interviews and had complete mortality data. Exclusion criteria were: (1) age < 18 years; (2) non‐asthmatic individuals; (3) missing DII or HEI‐2015 dietary data; and (4) lack of follow‐up. The final sample size was 609 participants (Figure 1).

### 2.2 Definition of ACO

A diagnosis of asthma was assigned to participants who fulfilled at least one of the following conditions:

1. A prior medical diagnosis of asthma provided by a physician or another qualified health professional.
2. A documented use of anti‐asthmatic medication.
3. For participants under 40 years of age with no history of smoking, chronic bronchitis, or emphysema, the use of specific respiratory medications. These included selective phosphodiesterase‐4 inhibitors, mast cell stabilizers, leukotriene modifiers, or inhaled corticosteroids.

A diagnosis of COPD was assigned to participants who met at least one of the following conditions:

1. A post‐bronchodilator spirometry result showing a forced expiratory volume in 1 s (FEV1) to forced vital capacity (FVC) ratio of less than 0.70.
2. A prior medical diagnosis of emphysema provided by a physician or another qualified health professional.
3. For participants aged 40 years or older with a history of smoking and chronic bronchitis, the use of specific respiratory medications. These included selective phosphodiesterase‐4 inhibitors, mast cell stabilizers, leukotriene modifiers, or inhaled corticosteroids.

Participants who met at least one criterion for both asthma and COPD were classified as having ACO.

### 2.3 Dietary Assessment

The DII is a scientifically validated, quantitative instrument developed from peer‐reviewed literature to evaluate the inflammatory potential of an individual's diet. It incorporates 45 distinct dietary components—such as macronutrients, micronutrients, vitamins, minerals, and bioactive compounds like phytochemicals—to generate a standardized score that reflects the diet's capacity to modulate systemic inflammation. This score is calibrated using established inflammatory biomarkers, including interleukin‐6 (IL‐6) and C‐reactive protein (CRP), and others. The DII produces values on a continuous spectrum, where negative scores signify anti‐inflammatory properties and positive scores indicate pro‐inflammatory effects. Higher DII values are associated with an increased propensity to promote inflammatory responses (Shivappa et al. 2014).

The HEI‐2015 is an assessment tool used to measure diet quality in relation to the 2015–2020 Dietary Guidelines for Americans. The HEI‐2015 comprises 13 components, with a total score of 100 points, divided into adequacy components (9 items, e.g., whole grains, fruits, vegetables) and moderation components (4 items, e.g., refined grains, sodium, added sugars). Higher scores indicate a healthier diet that better aligns with nutritional recommendations (Krebs‐Smith et al. 2018). More detailed information is presented in the Methods section in the Supplement.

### 2.4 Ascertainment of Mortality

The NHANES obtains mortality follow‐up data by linking with the National Death Index (NDI). The NDI, maintained by the NCHS, is a centralized database that records mortality information for U.S. residents based on death certificates, including death status, date of death, and cause of death. NHANES generates mortality data through probabilistic matching of participants' personal information (e.g., name, Social Security Number, date of birth, sex) with the NDI database. Mortality data for the NHANES 2011–2018 cycles were tracked up to the latest available NDI year, December 31, 2019.

### 2.5 Statistical Analysis

To derive nationally representative population estimates, sample weighting was applied. The relationships of the DII and the HEI‐2015 with all‐cause mortality were examined using Cox proportional hazards regression models. Both indices were analyzed as continuous variables and, to explore potential threshold effects, as quartile‐based categorical variables.

To holistically assess the interplay between diet quality and inflammatory potential, participants were classified into four distinct dietary pattern groups based on a median split of their DII and HEI‐2015 scores: (1) unhealthy pro‐inflammatory diet (*n* = 228), (2) healthy pro‐inflammatory diet (*n* = 105), (3) unhealthy anti‐inflammatory diet (*n* = 91), and (4) healthy anti‐inflammatory diet (*n* = 185).

Multivariable analyses were meticulously adjusted for a comprehensive set of potential confounders, including sociodemographic factors (age, sex, race/ethnicity, marital status, household income, education level), lifestyle variables (smoking status, alcohol consumption, physical activity), clinical characteristics (body mass index [BMI], diabetes, hypertension, estimated glomerular filtration rate [eGFR]), and nutritional factors (dietary supplement use, total energy intake). The specific methodologies for covariate assessment are elaborated upon in the Supporting Information. Furthermore, to characterize any potential non‐linear relationships, restricted cubic spline analyses were employed for both the DII and HEI‐2015 in relation to mortality risk.

To evaluate the robustness of the primary findings and to investigate heterogeneity across population subgroups, a series of stratified analyses were performed. These analyses were stratified by age (< 60 vs. ≥ 60 years), sex, race/ethnicity, smoking status, alcohol consumption, hypertension status, and diabetes status. All primary analytical procedures were repeated within these strata. In a sensitivity analysis intended to mitigate potential reverse causation, deaths occurring within the initial 2 years of follow‐up were excluded.

Finally, to identify the specific dietary components most strongly predictive of mortality, a Least Absolute Shrinkage and Selection Operator (LASSO) regression analysis was conducted on the constituent food and nutrient items of the DII and HEI‐2015. A predictive nomogram was subsequently developed based on the variables selected by the LASSO procedure. The discriminative performance of this nomogram was quantified by the area under the receiver operating characteristic curve (AUC). Based on the median risk score from the nomogram, the cohort was dichotomized into high‐risk and low‐risk groups. Survival disparities between these groups were then visualized and compared using Kaplan–Meier survival curves.

## 3 Results

### 3.1 Patient Characteristics

Participants in four dietary groups—unhealthy and pro‐inflammatory (UPI, *N* = 228), healthy and pro‐inflammatory (HPI, *N* = 105), unhealthy and anti‐inflammatory (UAI, *N* = 91), and healthy and anti‐inflammatory (HAI, *N* = 185)—showed significant differences in demographics, lifestyle, and health (Table 1). HPI was oldest (median 62 years) and mostly female (79.91%), while HAI had the most Non‐Hispanic Whites (81.54%), married (71.48%), and college‐educated individuals (74.01%), with low smoking (11.81%). UPI had high smoking (46.91%) and BMI (31.81 kg/m^2^), while UAI had the highest energy intake (2687.22 kcal). Education, income, and supplement use varied significantly (*p* < 0.05), but physical activity and hypertension did not.

**TABLE 1:** Characteristic of study sample.

Characteristic	Unhealthy and pro‐inflammatory diet *N* = 228	Healthy and pro‐inflammatory diet *N* = 105	Unhealthy and anti‐inflammatory diet *N* = 91	Healthy and anti‐inflammatory diet *N* = 185	*p*
Age, years	55.00 (49.00, 65.00)	62.00 (54.00, 74.00)	59.00 (52.00, 68.00)	62.00 (54.00, 68.00)	0.003
Sex					
Male	87 (33.43)	26 (20.09)	57 (60.09)	103 (47.74)	< 0.001
Female	141 (66.57)	79 (79.91)	34 (39.91)	82 (52.26)	
Race/ethnicity					
Non‐Hispanic Black	46 (12.74)	21 (9.90)	17 (5.76)	29 (5.07)	0.01
Non‐Hispanic White	135 (73.55)	54 (71.74)	59 (80.41)	111 (81.54)	
Mexican American	8 (1.45)	11 (6.81)	2 (0.96)	15 (2.45)	
Other Hispanic	18 (4.67)	13 (6.09)	1 (0.35)	15 (3.00)	
Other race—including multi‐racial	21 (7.58)	6 (5.46)	12 (12.52)	15 (7.94)	
Marital					
Never married	27 (9.85)	8 (5.27)	10 (7.15)	11 (9.17)	0.01
Married/living with partner	100 (50.59)	44 (52.43)	48 (69.96)	119 (71.48)	
Divorced/widowed/separated	101 (39.56)	53 (42.29)	33 (22.89)	55 (19.35)	
Family income poverty ratio	1.63 (0.88, 3.39)	1.61 (0.93, 2.81)	2.06 (1.47, 2.81)	3.40 (1.55, 5.00)	< 0.001
Education					
Less than high school	26 (7.23)	16 (10.01)	9 (7.15)	16 (3.11)	0.004
High school or equivalent	108 (43.17)	51 (47.22)	41 (45.28)	51 (22.87)	
College and above	94 (49.59)	38 (42.77)	41 (47.56)	118 (74.01)	
Smoking status					
Never	33 (14.86)	23 (20.38)	19 (18.06)	55 (34.93)	< 0.0001
Former	88 (38.23)	46 (45.26)	36 (41.23)	101 (53.27)	
Now	107 (46.91)	36 (34.36)	36 (40.70)	29 (11.81)	
Drinking status					
Never	17 (7.38)	9 (4.08)	3 (1.28)	13 (10.34)	0.05
Former	77 (28.58)	33 (25.71)	30 (32.59)	44 (15.81)	
Mild	66 (30.17)	40 (47.89)	29 (38.19)	80 (45.51)	
Moderate	29 (13.21)	7 (8.55)	10 (14.20)	31 (18.38)	
Heavy	39 (20.66)	16 (13.76)	19 (13.74)	17 (9.96)	
MET scores, min/week	2880.00 (1200.00, 4793.44)	2400.00 (1200.00, 4080.00)	2982.17 (1440.00, 4558.55)	3020.00 (1200.00, 5434.56)	0.65
DM					
No	146 (65.89)	66 (69.71)	60 (65.15)	122 (77.75)	0.17
Yes	82 (34.11)	39 (30.29)	31 (34.85)	63 (22.25)	
Hypertension					
No	69 (34.84)	35 (43.23)	34 (53.26)	54 (35.07)	0.17
Yes	159 (65.16)	70 (56.77)	57 (46.74)	131 (64.93)	
Use of supplement					
No	110 (45.75)	42 (32.79)	38 (33.93)	48 (21.54)	0.01
Yes	118 (54.25)	63 (67.21)	53 (66.07)	137 (78.46)	
BMI, kg m^2^	31.81 ± 0.61	31.54 ± 0.99	28.32 ± 1.14	30.72 ± 0.93	0.05
Energy, kcal	1638.76 ± 53.84	1329.67 ± 54.24	2687.22 ± 134.46	2063.56 ± 62.05	< 0.001

Abbreviations: DM, diabetes mellitus; MET, metabolic equivalent of task.

### 3.2 The Synergistic Effect of Two Different Dietary Patterns on Mortality

Table 2 shows that healthier diets (higher HEI‐2015 scores) are linked to lower mortality risk in asthma patients, with a significant protective effect in the highest quartile (HR = 0.61, Model 2). More pro‐inflammatory diets (higher DII scores) increase mortality risk (HR = 1.45, Model 2). The combination of a healthy (high HEI‐2015) and anti‐inflammatory (low DII) diet was associated with the lowest all‐cause mortality risk (HR = 0.68, 95% CI: 0.47–0.98 in fully adjusted Model 2), compared with the reference group of an unhealthy and pro‐inflammatory diet, with a significant dose–response trend across the four dietary pattern groups (*p*‐for‐trend = 0.044).

**TABLE 2:** Relationship between dietary habits and mortality in ACO patients.

Outcomes	Crude model	Model 1	Model 2
	HR (95% CI)	HR (95% CI)	HR (95% CI)
HEI‐2015			
Continuous	0.99 (0.99, 1.00)	0.99 (0.98, 1.00)	0.98 (0.97, 0.99)
Categories			
Q1 (≤ 44.64)	Ref.	Ref.	Ref.
Q2 (44.64, 52.35)	1.20 (0.88, 1.64)	1.04 (0.72, 1.50)	0.96 (0.67, 1.38)
Q3 (52.35, 61.29)	0.97 (0.67, 1.41)	0.95 (0.67, 1.34)	0.78 (0.56, 1.09)
Q4 (> 61.29)	0.81 (0.63, 1.06)	0.68 (0.46, 1.00)	0.61 (0.40, 0.95)
*p* for trend	0.066	0.038	0.013
DII			
Continuous	1.02 (0.96, 1.09)	1.04 (0.96, 1.12)	1.13 (1.04, 1.22)
Categories			
Q1 (≤ 0.62)	Ref.	Ref.	Ref.
Q2 (0.62, 1.83)	0.91 (0.67, 1.23)	0.92 (0.69, 1.24)	1.09 (0.74, 1.61)
Q3 (1.83, 3.07)	1.05 (0.74, 1.48)	1.06 (0.70, 1.60)	1.22 (0.84, 1.79)
Q4 (> 3.07)	1.06 (0.81, 1.38)	1.11 (0.80, 1.56)	1.45 (1.03, 2.04)
*p* for trend	0.546	0.487	0.03
Composition effect			
Categories			
Unhealthy and pro‐inflammatory diet	Ref.	Ref.	Ref.
Healthy and pro‐inflammatory diet	1.05 (0.77, 1.45)	0.99 (0.70, 1.41)	0.94 (0.64, 1.38)
Unhealthy and anti‐inflammatory diet	1.24 (0.82, 1.87)	1.19 (0.75, 1.88)	1.25 (0.82, 1.90)
Healthy and anti‐inflammatory diet	0.82 (0.61, 1.09)	0.76 (0.53, 1.09)	0.68 (0.47, 0.98)
*p* for trend	0.241	0.225	0.044

*Note:* crude model: no covariates were adjusted. Model 1: adjusted covariates for model 1 included age, gender, race, marital status, family income level, and educational level. Model 2: adjusted covariates for model 2 included the covariates for model 1 plus smoking status, alcohol intake, physical activity, BMI, diabetes, hypertension, use of supplements, total Kal intake and eGFR.

Abbreviations: 95% CI, 95% confidence interval; DII, Dietary Inflammatory Index; HEI, Healthy Eating Index; HR, hazard ratio.

**FIGURE 2:**
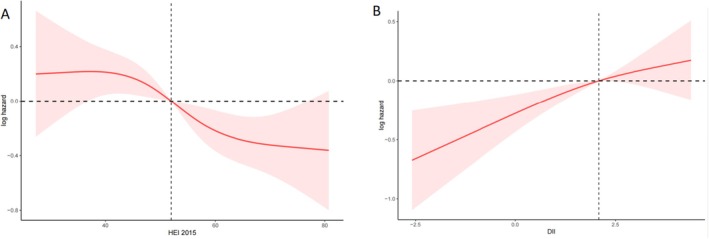
Restricted cubic spline (RCS) curves illustrating the relationship between dietary indices and long‐term mortality in ACO patients. (A) Healthy eating index‐2015 (HEI‐2015). (B) Dietary inflammatory index (DII).

Furthermore, restricted cubic spline (RCS) analysis corroborated these findings, revealing no evidence of significant non‐linear relationships between either the HEI‐2015, or DII scores and long‐term mortality risk (Figure 2).

### 3.3 Subgroup Analyses

Table 3 presents the stratified analysis results of the association between the HEI‐2015 and mortality in ACO patients (Model 2, with Q1 as the reference). A healthy diet was more significantly associated with lower mortality risk in mild drinkers and patients without hypertension.

**TABLE 3:** Stratified analyses of the relationships of dietary patterns and mortality among ACO patients.

Characteristics	Unhealthy and pro‐inflammatory diet *N* = 228	Healthy and pro‐inflammatory diet *N* = 105	Unhealthy and anti‐inflammatory diet *N* = 91	Healthy and anti‐inflammatory diet *N* = 185	*p* for trend	*p* for interaction
Age						
< 60	Ref.	1.00 (0.56, 1.78)	1.29 (0.79, 2.12)	0.63 (0.35, 1.11)	0.15	0.99
≥ 60	Ref.	0.75 (0.38, 1.49)	1.16 (0.52, 2.56)	0.65 (0.32, 1.35)	0.24	
Gender						
Male	Ref.	1.07 (0.50, 2.25)	1.47 (0.89, 2.44)	0.65 (0.33, 1.27)	0.22	0.76
Female	Ref.	1.02 (0.69, 1.49)	1.62 (0.78, 3.36)	0.71 (0.40, 1.26)	0.31	
Race/ethnicity						
Non‐Hispanic Black	Ref.	2.33 (0.87, 6.26)	0.64 (0.23, 1.75)	0.58 (0.27, 1.25)	0.09	0.01
Non‐Hispanic White	Ref.	0.99 (0.62, 1.59)	1.70 (1.03, 2.79)	0.68 (0.43, 1.05)	0.09	
Mexican American	Ref.	0.20 (0.01, 2.81)	0.93 (0.01, 109.68)	0.01 (0.00, 3.86)	0.03	
Other Hispanic	Ref.	0.12 (0.00, 9.94)	0.00 (0.00, 0.59)	0.49 (0.03, 9.36)	0.91	
Other race—including multi‐racial	Ref.	0.20 (0.00, 8.01)	1.66 (0.33, 8.35)	2.35 (0.61, 9.09)	0.4	
Smoke status						
Never	Ref.	1.30 (0.42, 4.05)	1.47 (0.49, 4.37)	0.78 (0.37, 1.66)	0.3	0.12
Former	Ref.	0.74 (0.37, 1.47)	1.93 (0.84, 4.45)	0.68 (0.34, 1.34)	0.26	
Now	Ref.	1.12 (0.63, 1.98)	0.72 (0.39, 1.31)	0.50 (0.25, 1.01)	0.07	
Drinking status						
Never	Ref.	0.10 (0.00, 3101.88)	9.50 (0.95, 95.01)	4.76 (0.07, 314.46)	0.24	0.3
Former	Ref.	1.39 (0.49, 3.94)	1.47 (0.60, 3.58)	0.91 (0.44, 1.87)	0.92	
Mild	Ref.	0.78 (0.37, 1.63)	1.26 (0.55, 2.86)	0.42 (0.25, 0.71)	< 0.001	
Moderate	Ref.	1.62 (0.44, 5.99)	3.55 (0.84, 15.0)	0.71 (0.33, 1.55)	0.43	
Heavy	Ref.	1.12 (0.46, 2.69)	0.88 (0.22, 3.53)	0.44 (0.07, 2.90)	0.46	
Hypertension						
No	Ref.	0.90 (0.47, 1.69)	1.18 (0.61, 2.31)	0.37 (0.16, 0.83)	0.02	0.5
Yes	Ref.	0.93 (0.58, 1.50)	1.29 (0.69, 2.41)	0.69 (0.43, 1.12)	0.15	
DM						
No	Ref.	1.14 (0.71, 1.82)	1.19 (0.71, 2.00)	0.78 (0.52, 1.17)	0.2	0.35
Yes	Ref.	0.68 (0.33, 1.40)	1.28 (0.55, 3.01)	0.58 (0.29, 1.17)	0.17	

*Note:* The adjusted covariates encompassed age, gender, race, marital status, family income level, educational attainment, smoking status, alcohol consumption, physical activity, diabetes, hypertension, use of supplements, total kcal intake and eGFR, excluding the stratified variables.

Abbreviation: DM, diabetes mellitus.

### 3.4 LASSO Regression Analysis on Dietary Factors and Mortality Risk in ACO Patients

**FIGURE 3:**
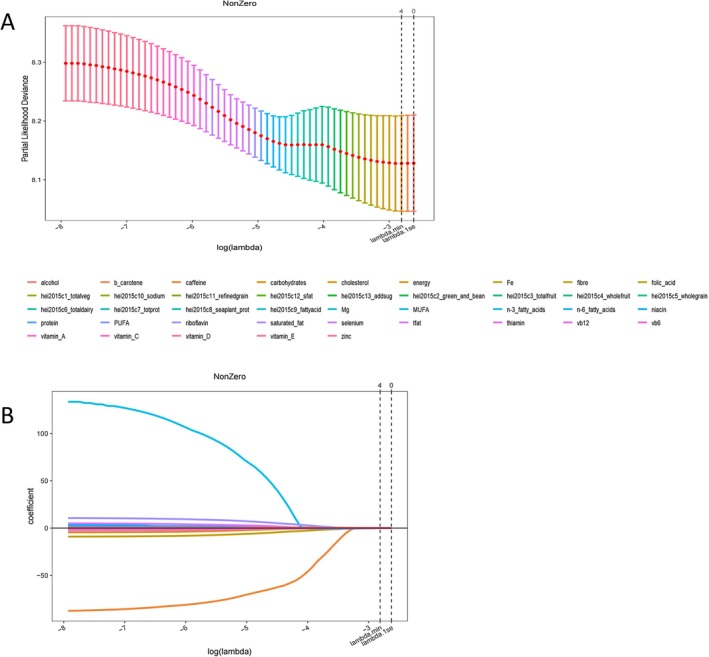
LASSO regression analysis for screening dietary factors linked to ACO mortality. (A) Trajectory of coefficient shrinkage across 41 dietary variables. (B) Ten‐fold cross‐validation curve.

**FIGURE 4:**
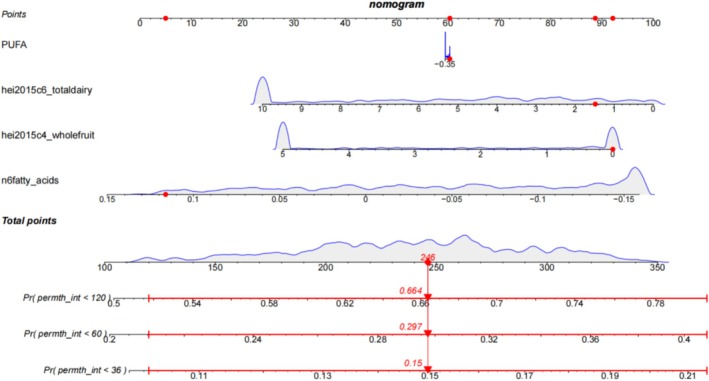
Prognostic nomogram incorporating dietary components identified by LASSO regression.

Using LASSO regression, we identified key HEI‐2015 components—PUFA, total dairy, whole fruit, and n‐6 fatty acids—linked to mortality, as shown in Figure 3. These components informed a prognostic nomogram for asthma patients to predict mortality risk (Figure 4).

**FIGURE 5:**
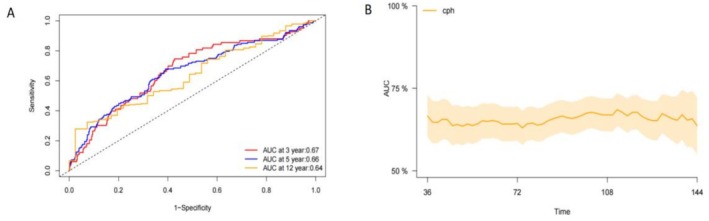
Predictive performance of the nomogram model for ACO mortality. (A) ROC curve for all‐cause mortality prediction. (B) Time‐dependent ROC analysis for overall survival.

The nomogram's predictive accuracy was evaluated using receiver operating characteristic (ROC) curves and time‐dependent AUC analyses, detailed in Figure 5. Figure 5A presents ROC curves with AUCs of 0.67 at 3 years, 0.66 at 5 years, and 0.64 at 12 years, indicating moderate and declining discriminative ability over time. Figure 5B shows time‐dependent AUC values ranging from 50% to 75% over 36 to 144 months, with a downward trend suggesting reduced model accuracy with longer follow‐up.

**FIGURE 6:**
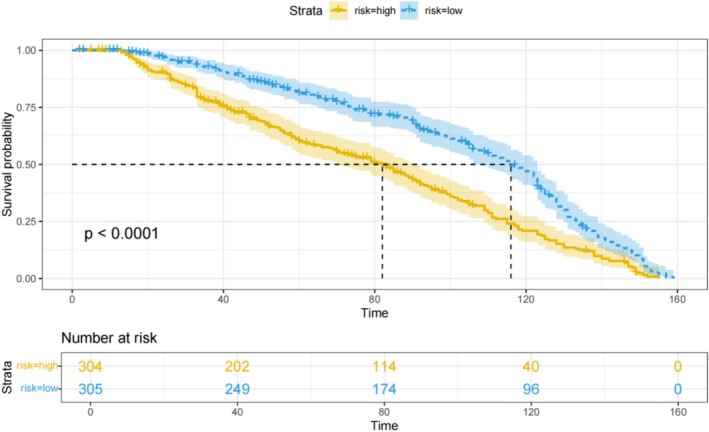
Kaplan–Meier estimates comparing mortality risk in patient subgroups identified by LASSO regression modeling.

Figure 6 displays Kaplan–Meier survival curves for asthma patients, stratified by the median of the nomogram risk score into high‐risk (*n* = 304) and low‐risk (*n* = 305) cohorts. The analysis reveals a markedly greater survival probability in the low‐risk group (represented in blue) relative to the high‐risk group (shown in yellow), with the curves exhibiting persistent divergence throughout the 0–160 month follow‐up duration (*p* < 0.0001).

### 3.5 Sensitivity Analysis

Table 4 shows Cox regression results for dietary habits and mortality in asthma patients, excluding deaths within 1 year. Compared to the unhealthy and pro‐inflammatory diet (reference), the healthy and pro‐inflammatory diet had an HR of 0.94 (95% CI: 0.64–1.38), and the unhealthy and anti‐inflammatory diet had an HR of 1.25 (95% CI: 0.83–1.88), both non‐significant. The healthy and anti‐inflammatory diet had a significant HR of 0.68 (95% CI: 0.47–0.98, *p* < 0.05). The *p* for trend was 0.045, indicating a significant trend toward lower mortality with healthier, anti‐inflammatory diets.

**TABLE 4:** Cox regression analysis of dietary habits and mortality in ACO patients excluding deaths within 1 year.

Outcomes	Crude model	Model 1	Model 2
	HR (95% CI)	HR (95% CI)	HR (95% CI)
Composition effect			
Categories			
Unhealthy and pro‐inflammatory diet	Ref.	Ref.	Ref.
Healthy and pro‐inflammatory diet	1.05 (0.73, 1.51)	0.99 (0.68, 1.44)	0.94 (0.64, 1.38)
Unhealthy and anti‐inflammatory diet	1.24 (0.83, 1.85)	1.19 (0.78, 1.82)	1.25 (0.83, 1.88)
Healthy and anti‐inflammatory diet	0.82 (0.58, 1.16)	0.76 (0.52, 1.11)	0.68 (0.47, 0.98)
*p* for trend	0.240	0.220	0.045

## 4 Discussion

This study highlights the significant impact of dietary patterns on mortality in asthma patients with ACO, as assessed by the HEI‐2015 and DII. Healthier diets with lower inflammatory potential were consistently linked to reduced mortality risk, with the strongest protective effect observed for diets combining high quality and anti‐inflammatory properties, particularly after adjusting for confounders and excluding early deaths. Restricted cubic spline analyses confirmed linear associations between dietary scores and mortality, showing no significant non‐linearity. LASSO regression identified key dietary components, including PUFA, total dairy, whole fruit, and n‐6 fatty acids, which were used to develop a prognostic nomogram with moderate predictive accuracy, as shown in receiver operating characteristic curves. Kaplan–Meier survival curves demonstrated markedly better survival for patients with lower dietary risk scores. These findings are consistent with prior research linking healthy, anti‐inflammatory diets to improved respiratory outcomes and underscore the potential of targeted nutritional strategies to enhance survival in ACO patients.

Our results align with emerging evidence linking dietary patterns to respiratory health outcomes. Prior studies have shown that adherence to high‐quality diets, such as the Mediterranean or DASH patterns, is associated with reduced COPD risk and improved lung function (Parvizian et al. 2020; Wen et al. 2023; Ardestani et al. 2017; Fischer et al. 2019). For example, a comprehensive population‐based study in the United States identified a statistically significant protective effect linked to adherence to a “prudent” dietary pattern, characterized by high consumption of fruits, vegetables, whole grains, poultry, low‐fat dairy, and oily fish (Brigham et al. 2018). The findings specifically demonstrated that this diet was associated with a reduced incidence and severity of cough, aligning with the adequacy components of the HEI‐2015. Similarly, elevated DII scores have been correlated with increased inflammatory biomarkers like CRP and IL‐6, which exacerbate airway inflammation in chronic respiratory diseases (Shivappa et al. 2014). In the context of ACO, which combines features of asthma (e.g., eosinophilic inflammation) and COPD (e.g., neutrophilic inflammation and irreversible airflow obstruction), our findings extend these observations by highlighting the additive benefits of combining anti‐inflammatory and nutrient‐dense diets. This synergy may explain the stronger mortality reduction in the healthy anti‐inflammatory group compared to other combinations, consistent with Nygaard et al.'s report of lower inflammatory markers in asthma patients with improved diet quality (Nygaard et al. 2021).

The biological mechanisms underlying these associations likely involve modulation of systemic inflammation and oxidative stress, key drivers of ACO progression (Fouka et al. 2022). Anti‐inflammatory diets low in DII (e.g., rich in antioxidants from whole fruits) can attenuate pro‐inflammatory pathways, such as NF‐κB activation, thereby reducing exacerbations and lung function decline (Zhai et al. 2018). PUFA and n‐6 fatty acids, identified via LASSO, may contribute through eicosanoid metabolism, promoting resolution of inflammation (Liput et al. 2021; Wei et al. 2021). Dairy products, another selected component, provide bioactive peptides and vitamin D, which support immune regulation and bone health in patients often treated with corticosteroids (Li et al. 2020; Bikle 2022). Whole fruits offer fiber and polyphenols that enhance gut microbiome diversity, indirectly benefiting respiratory health via the gut‐lung axis (Katsirma et al. 2021; Wang et al. 2023). These components' inclusion in our nomogram suggests targeted interventions could personalize nutritional strategies for ACO patients.

The present findings underscore the potential value of nutritional interventions as a modifiable component in ACO management. Given the complexity of dietary counseling—particularly in patients with chronic respiratory diseases who may have comorbidities, medication‐related nutritional needs (e.g., corticosteroids), swallowing difficulties, or low appetite—such interventions are most effectively delivered by registered dietitians with specialized training in respiratory nutrition or asthma/COPD care. Multidisciplinary teams including pulmonologists, respiratory therapists, and dietitians are recommended worldwide to provide individualized, evidence‐based nutritional education and monitoring, thereby maximizing adherence and clinical benefit.

Strengths of this study include its large, nationally representative sample, comprehensive adjustment for confounders (e.g., sociodemographics, lifestyle, and clinical factors), and use of validated dietary indices (HEI‐2015 and DII) derived from 24‐h recalls. The integration of LASSO regression for variable selection and nomogram development adds prognostic utility, potentially aiding clinical risk stratification. Mortality ascertainment via linkage to the National Death Index ensures reliable outcomes.

However, limitations warrant consideration. First, the cross‐sectional assessment of diet at baseline may not capture long‐term patterns, introducing measurement error from self‐reported recalls. Second, while we adjusted for numerous covariates, residual confounding (e.g., from air pollution exposure or medication adherence) cannot be ruled out. Third, ACO definition relied on self‐reported diagnoses and spirometry where available, potentially leading to misclassification, though our criteria align with established guidelines. Fourth, the nomogram's moderate AUC indicates room for improvement, possibly by incorporating biomarkers or genetic data. Finally, the observational design precludes causality, randomized trials are needed to confirm dietary interventions' efficacy.

## 5 Conclusion

In conclusion, our study provides compelling evidence that optimizing diet quality (high HEI‐2015) and reducing dietary inflammatory potential (low DII) synergistically lowers all‐cause mortality in patients with ACO. These findings highlight the potential value of nutritional interventions emphasizing whole fruits, dairy, and healthy fats (e.g., PUFA and n‐6 fatty acids) in this high‐risk population. To translate these observational associations into clinical practice, nutritional counseling should be incorporated into ACO management as part of a multidisciplinary approach, ideally led or delivered by registered dietitians with expertise in respiratory nutrition or asthma/COPD care. Future interventional trials and implementation studies are needed to evaluate the efficacy, feasibility, and long‐term impact of targeted dietary strategies, including dietitian‐led programs, in improving survival and quality of life for patients with ACO.

## Author Contributions

Jixiang Li, Jia Yi, and Jingxian Tang contributed to the study design, data analysis, and initial manuscript drafting. Tong Feng Rongrong Guo and Yuan Wang provided critical revisions to the document and approved the final submitted version of the manuscript. All authors reviewed and endorsed the manuscript prior to submission.

## Funding

The authors have nothing to report.

## Disclosure

During the preparation of this work the author(s) used GPT‐4 in order to improve language, readability, and clarity of text passages. After using this tool, the author(s) carefully reviewed and edited the content as needed and take full responsibility for the content of the publication. No AI technology was used to generate scientific content, data, analyses, figures, results, or conclusions. All claims, interpretations, and intellectual contributions are those of the authors.

## Ethics Statement

The ethics review board of the National Center for Health Statistics approved all NHANES protocols, and written informed consent was obtained from all participants or their proxies for those under 18 years of age. The study was conducted in accordance with the ethical standards outlined in the 1964 Declaration of Helsinki and its subsequent amendments.

## Consent

The authors have nothing to report.

## Conflicts of Interest

The authors declare no conflicts of interest.

## Supporting information


**Data S1:** fsn371657‐sup‐0001‐Supinfo.docx.

## Data Availability

The survey data are publicly available on the internet for data users and researchers throughout the world (www.cdc.gov/nchs/nhanes/).
