# Supplementary material for: Diet Quality, Dietary Inflammatory Potential, and All‐Cause Mortality in U.S. Adults With Asthma‐COPD Overlap
Source: Food Sci Nutr. 2026 Jul 8;14(7):e71657. doi: 10.1002/fsn3.71657 (PMC13342702; doi:10.1002/fsn3.71657)
Supplement: Supplementary file 1 — Data S1: fsn371657‐sup‐0001‐Supinfo.docx. [file FSN3-14-e71657-s001.docx]

**Supplementary Material**

**“Nutrition as a Healing Approach for Asthma Wellness: How HEI-2015 Scores Influence Asthma Mortality”**

**Dietary assessment**

The NHANES employs a robust two-phase 24-Hour Dietary Recall (24-HDR) methodology to collect detailed dietary intake data. The initial in-person interview, conducted at the Mobile Examination Center (MEC) by trained professionals, follows a standardized protocol to ensure accurate recall of all foods and beverages consumed in the preceding 24-hour period (midnight to midnight). A follow-up telephone interview, administered 3–10 days later, captures intra-individual variability in dietary patterns. NHANES utilizes the U.S. Department of Agriculture’s (USDA) Automated Multiple-Pass Method (AMPM), a structured five-step process comprising an initial unprompted recall (Quick List), targeted probes for omitted items (Forgotten Foods Probe), contextual details on eating occasions (Time and Occasion), specifics on portion sizes, preparation, and additives (Detailed Description), and a final verification of the dietary record (Final Review). To enhance portion size accuracy, participants use standardized tools such as measuring cups, three-dimensional food models, and two-dimensional visual aids. For this study, dietary data from NHANES cycles 2007–2018 were analyzed. To address day-to-day intake variability, the mean of both recalls was calculated for participants with complete two-day data, while single-day intake values were used for those with only one recall^(1-4)^.

A single 24-hour recall does not fully capture long-term usual dietary patterns at the individual level due to day-to-day variation; however, the use of one or two recalls is a standard, validated approach in NHANES for estimating population-level diet quality, nutrient intakes, and inflammatory potential, particularly when survey weights are applied to account for sampling design, non-response, and oversampling . Averaging two recalls, as done here whenever possible, substantially reduces intra-individual variability compared to a single day.

This methodology has been successfully applied in numerous NHANES-based studies examining associations between HEI-2015, DII, and chronic health outcomes. Usual intake distributions can be further refined by adjusting for within-person variation using established statistical methods (e.g., NCI usual intake macros), though such advanced adjustments were not required for the primary categorical and quartile-based analyses in this study.

Detailed information on NHANES dietary data collection, processing, weighting, and limitations is provided in the official NHANES Dietary Data Documentation (available at www.cdc.gov/nchs/nhanes/)

**Healthy Eating Index**

The HEI-2015 is a validated dietary assessment tool comprising 13 components, categorized into adequacy and moderation components. The adequacy components include total fruits, whole fruits, total vegetables, greens and beans, whole grains, dairy, total protein foods, seafood and plant-based proteins, and the fatty acid ratio (polyunsaturated and monounsaturated fatty acids relative to saturated fatty acids)^(5)^.

The HEI-2015 scores were calculated using the standardized scoring algorithm developed by the National Cancer Institute (NCI). Dietary intake data, sourced from the Food Patterns Equivalents Database (FPED) and the Food and Nutrient Database for Dietary Studies (FNDDS), were normalized to a standard energy intake of 1,000 kilocalories (kcal) to enable consistent comparisons across components, such as fruit consumption (measured in cup equivalents) and sodium intake (quantified in milligrams). Each of the 13 HEI-2015 components was assessed against predefined scoring criteria; for example, the whole grains component received 0 points for an intake of 0 ounce equivalents per 1,000 kcal and a maximum of 10 points for consumption meeting or exceeding 1.5 ounce equivalents per 1,000 kcal. These individual component scores were then aggregated to produce a total HEI-2015 score, with higher composite scores indicating greater adherence to dietary guidelines and superior overall diet quality.

**Dietary Inflammatory Index**

The DII is an empirically validated scoring system designed to quantify the inflammatory potential of an individual's dietary intake, elucidating the link between diet and systemic inflammation. A comprehensive literature review identified 45 key dietary parameters significantly associated with inflammatory biomarkers, encompassing macronutrients (e.g., fats, carbohydrates, proteins), micronutrients (e.g., vitamins A, C, D, E, zinc, magnesium), bioactive compounds (e.g., polyphenols, flavonoids), and specific food items (e.g., garlic, alcohol). In this study, 28 dietary components from the NHANES dataset were used to calculate the DII, including energy intake, macronutrients (carbohydrates, proteins, total fats, saturated fats, monounsaturated fats, polyunsaturated fatty acids [n-3 and n-6], cholesterol), micronutrients (vitamins A, B1, B2, B6, B12, C, D, E, niacin, folate, iron, magnesium, selenium, zinc), and additional factors (caffeine, alcohol)^(6)^.

The DII calculation involves a systematic process that starts with collecting dietary intake data using validated tools, such as food frequency questionnaires, 24-hour dietary recalls, or food records, to quantify consumption of specific dietary parameters. These intake values are then standardized into Z-scores, reflecting the deviation of an individual’s intake from global mean intake levels, enabling uniform comparisons across diverse populations and dietary components. Subsequently, Z-scores are transformed using a sigmoid function to convert them into percentiles, constraining values within a standardized range from -1 (indicating maximal anti-inflammatory potential) to +1 (indicating maximal pro-inflammatory potential).

**Assessment of Covariates**

The NHANES collected covariates through structured questionnaires, clinical examinations, and laboratory assessments to adjust for potential confounding factors in mortality analyses. These covariates included demographic factors: age (continuous, in years), sex (self-reported as male or female), race/ethnicity (categorized as Non-Hispanic White, Non-Hispanic Black, Hispanic, or Other), and marital status (married, widowed, divorced, separated, never married, or cohabiting); socioeconomic indicators: family income, assessed via the poverty income ratio (PIR), and educational attainment (less than high school, high school graduate, or college or higher); lifestyle and behavioral factors: smoking status (never, former, or current smoker, based on self-reported cigarette use), alcohol consumption (none, mild, moderate, or heavy, derived from dietary interviews), and physical activity level, evaluated by self-reported exercise frequency, duration, and intensity, expressed in Metabolic Equivalent of Task (MET)-minutes per week; and clinical and metabolic parameters: diabetes status (defined by self-reported diagnosis, fasting glucose ≥126 mg/dL, HbA1c ≥6.5%, or antidiabetic medication use), hypertension (determined by self-reported diagnosis, average blood pressure ≥140/90 mmHg from up to three readings, or antihypertensive drug use), dietary supplement use (self-reported intake of vitamins, minerals, or other supplements in the past 30 days), total daily caloric intake (kcal/day, estimated from 24-hour dietary recalls), and estimated glomerular filtration rate (eGFR), calculated using the Chronic Kidney Disease Epidemiology Collaboration (CKD-EPI) equation based on serum creatinine, age, sex, and race.

Reference

1. Ahluwalia N, Dwyer J, Terry A et al. (2016) Update on NHANES Dietary Data: Focus on Collection, Release, Analytical Considerations, and Uses to Inform Public Policy. Adv Nutr 7, 121-134.

2.Ahluwalia N, Dwyer J, Terry A, Moshfegh A, Johnson C. Update on NHANES Dietary Data: Focus on Collection, Release, Analytical Considerations, and Uses to Inform Public Policy. Adv Nutr. 2016;7(1):121-134. doi:10.3945/an.115.009258

3.Wang YB, Page AJ, Gill TK, Melaku YA. The association between diet quality, plant-based diets, systemic inflammation, and mortality risk: findings from NHANES. Eur J Nutr. 2023;62(7):2723-2737. doi:10.1007/s00394-023-03191-z

4.Jun S, Cowan AE, Dodd KW, et al. Association of food insecurity with dietary intakes and nutritional biomarkers among US children, National Health and Nutrition Examination Survey (NHANES) 2011-2016. Am J Clin Nutr. 2021;114(3):1059-1069. doi:10.1093/ajcn/nqab113

5.Reedy J, Lerman JL, Krebs-Smith SM et al. (2018) Evaluation of the Healthy Eating Index-2015. J Acad Nutr Diet 118, 1622-1633.

6.Mao Y, Weng J, Xie Q et al. (2024) Association between dietary inflammatory index and Stroke in the US population: evidence from NHANES 1999-2018. BMC Public Health 24, 50.
